# Active surveillance and risk prediction of piperacillin/tazobactam‑associated hypokalaemia: development and external validation of a clinical nomogram

**DOI:** 10.1007/s11096-026-02195-z

**Published:** 2026-07-24

**Authors:** Hehe Bai, Miao Guo, Nanbo Zheng, Xiaojing Nie

**Affiliations:** 1 Department of Pharmacy, Xi’ an Central Hospital, No. 185 Houzaimen, Xincheng, Xi’ an, 710003 Shaanxi China; 2 Department of Pharmacy, People’s Hospital of Tongchuan, Tongchuan, Shaanxi China; 3 Hospital President’s Office, Xi’ an Central Hospital, Xi’ an, Shaanxi China

**Keywords:** Adverse drug reaction, Hypokalaemia, Nomogram, Piperacillin/tazobactam, Pharmacovigilance, Risk prediction

## Abstract

**Introduction:**

Piperacillin/tazobactam (TZP) is widely used in hospitalised adults. Real‑world data suggest a higher risk of hypokalaemia than previously recognised.

**Aim:**

To investigate incidence, severity and risk factors of TZP‑induced hypokalaemia, and to develop and externally validate a nomogram for individualised risk prediction.

**Method:**

This retrospective study included hospitalised adults receiving TZP (2021–2025). TZP‑induced hypokalaemia was defined as serum potassium < 3.5 mmol/L ≥ 48 h after TZP initiation. Logistic regression identified predictors. A nomogram was constructed. Model performance was assessed by discrimination (area under the ROC curve, AUC), calibration (Hosmer‑Lemeshow test, calibration intercept/slope), Brier score and decision‑curve analysis (DCA), with internal and external validation.

**Results:**

Among 643 patients, 122 (19.0%) developed TZP‑induced hypokalaemia (mild: 78.7%; moderate: 16.4%; severe: 4.9%). Median time to onset was 5.0 days. Independent predictors were age ≥ 65 years (OR = 2.118, 95% CI 1.003–4.472, *p* = 0.049), body mass index (OR = 0.671, 95% CI 0.591–0.762, *p* < 0.001), intensive care unit admission (OR = 2.915, 95% CI 1.504–5.651, *p* = 0.002), daily dose (OR = 1.280, 95% CI 1.110–1.476, *p* < 0.001), and baseline serum potassium (OR = 0.123, 95% CI 0.056–0.271, *p* < 0.001). The nomogram showed good discrimination (training AUC 0.881, internal 0.876, external 0.813), calibration (Hosmer‑Lemeshow *p* > 0.05), Brier scores < 0.25 and positive net benefit on DCA.

**Conclusion:**

TZP‑induced hypokalaemia is common in hospitalised adults. A nomogram based on five readily available clinical variables may facilitate early identification of high‑risk patients, although prospective validation in diverse settings is required before clinical implementation.

**Supplementary Information:**

The online version contains supplementary material available at https://doi.org/10.1007/s11096-026-02195-z.

## Impact statements

- Nearly one in five hospitalised adults develops TZP‑induced hypokalaemia, highlighting the need for routine electrolyte monitoring during treatment.
- This validated nomogram uses five clinical predictors (age ≥65 years, body mass index [BMI], intensive care unit [ICU] admission, daily dose and baseline serum potassium) to identify high‑risk patients before TZP administration.
- The nomogram enables proactive electrolyte monitoring instead of post‑event intervention. However, prospective validation is still needed for widespread use.

## Introduction

Hypokalaemia (serum potassium < 3.5 mmol/L) affects up to 14% of outpatients and over 20% of hospitalised patients [1, 2]. Severe hypokalaemia is associated with muscle weakness, respiratory depression, paralytic ileus, heart failure, stroke and life-threatening cardiac arrhythmias [3–5]. It has also been independently linked to increased morbidity and mortality in patients with cardiovascular or renal conditions [6–8]. Most cases are drug-induced [9].

Piperacillin/tazobactam (TZP), a first‑line broad‑spectrum antibiotic, is widely used to treat severe bacterial infections [10, 11]. Early clinical trials underestimated its hypokalaemia risk, but emerging global real-world data confirm TZP-induced hypokalaemia as a prevalent inpatient adverse event [12]. A large pharmacovigilance study verified TZP as a high-risk β-lactam for hypokalaemia, independent of other electrolyte abnormalities [13]. TZP-related hypokalaemia usually develops 3–7 days after use (peak at day 5). Unmonitored potassium loss may lead to symptoms and require clinical intervention or drug adjustment [14]. Relevant drug authorities have updated product labelling to classify hypokalaemia as a serious adverse reaction [15].

The incidence of TZP-induced hypokalaemia ranges from 1.5%–12.6% in clinical trials to 17.9%–24.8% in real-world settings [16–19]. This adverse event often requires electrolyte correction or drug adjustment [9]. Known risk factors include advanced age, female gender, low baseline potassium, diarrhoea and high TZP doses [18–20].

Despite growing attention to this safety concern, few validated tools are available for individual risk assessment [13]. Nomograms are widely used for clinical risk prediction [21, 22]. They have been adopted for antibiotic dose optimisation but rarely for predicting drug-related adverse reactions [23, 24].

### Aim

This study developed and externally validated a nomogram for predicting TZP‑induced hypokalaemia to enable early risk identification and targeted monitoring.

## Method

### Study design and patient cohort

This retrospective cohort study at Xi’an Central Hospital included hospitalised adults (≥ 18 years) receiving TZP between 1 January 2021 and 31 December 2025. Hypokalaemia was defined as new-onset serum potassium < 3.50 mmol/L occurring ≥ 48 h after TZP initiation. The inclusion criteria were: (1) age ≥ 18 years; and (2) receipt of intravenous TZP for ≥ 2 days. Patients were excluded if they met any of the following criteria: (1) lack of documented serum potassium at baseline or after TZP treatment; (2) abnormal baseline serum potassium (< 3.5 or > 5.5 mmol/L); (3) use of potassium supplements or potassium-binding agents at baseline or during TZP therapy; (4) receipt of renal replacement therapy (e.g., haemodialysis, peritoneal dialysis, haemofiltration); (5) underlying conditions predisposing to hypokalaemia (e.g., vomiting, diarrhoea, metabolic alkalosis, gastric drainage, renal tubular acidosis, Cushing’s syndrome, primary hyperaldosteronism); (6) intravenous administration of other antimicrobials at baseline or during TZP therapy; or (7) incomplete clinical records.

Patient data were extracted from the hospital information system (HIS). All baseline and on‑treatment potassium levels were reviewed. Suspected cases were screened via the China Hospital Pharmacovigilance System (CHPS) [25]. Patients without CHPS alerts were assigned to the normokalaemia group. Two independent pharmacists assessed causality using the Naranjo scale. Scores ≥ 5 confirmed TZP‑induced hypokalaemia; scores < 5 were classified as negative [26]. Disagreements were resolved by a senior clinician. All laboratory results were manually reviewed to avoid missed cases and selection bias. The overall cohort was randomly divided into a training set and an internal validation set in a 7:3 ratio. The model was built using the training set and validated internally. For external validation, eligible patients were recruited from Tongchuan People’s Hospital between July and December 2025. Data were accessed on 26 January 2026 (Xi’an) and 10 February 2026 (Tongchuan).

### Information collection and definitions

Patient data were retrieved from the HIS via the CHPS and anonymised before extraction. Collected variables included demographics (age, sex, height, weight), clinical features (hospital stay, surgery, intensive care unit (ICU) admission), and comorbidities (hypertension, diabetes, coronary heart disease, cerebrovascular disease, chronic liver disease, chronic kidney disease). Baseline laboratory parameters measured before TZP initiation included serum potassium, calcium, sodium, phosphate; white blood cell count, haemoglobin, platelet count; total protein, albumin, aspartate aminotransferase (AST), alanine aminotransferase (ALT), serum creatinine, and blood urea nitrogen. Body mass index (BMI) was calculated. TZP exposure included daily dose, treatment course, and cumulative dose. Daily dose was recorded as the average daily piperacillin dose (g/day). TZP is a fixed‑ratio combination (8:1 piperacillin/tazobactam); all doses refer to piperacillin content only. Concurrent use of potassium‑affecting medications was documented, including agents that may elevate serum potassium (e.g., potassium‑sparing diuretics, non‑steroidal anti‑inflammatory drugs, β‑blockers, digoxin, angiotensin‑converting enzyme inhibitors, angiotensin II receptor blockers) and those that may lower serum potassium (e.g., potassium‑wasting diuretics, insulin, glucocorticoids).

Patients were divided into hypokalaemia and normokalaemia groups based on the occurrence of hypokalaemia after TZP treatment. Hypokalaemia was graded using CTCAE version 5.0: mild (3.0 to < 3.5 mmol/L), moderate (2.5 to < 3.0 mmol/L), or severe (< 2.5 mmol/L). Incidence, time to onset, and severity of TZP‑induced hypokalaemia were evaluated. Clinical characteristics and potential predictors were recorded and analysed for model development.

### Statistical analysis

All analyses were performed using SPSS 27.0 and R 4.3.2. Continuous variables were presented as mean ± standard deviation for normal distribution or median with interquartile range (IQR) for non-normal distribution. Categorical variables were described as frequencies and percentages. Between-group comparisons were performed using the Mann–Whitney *U* test (two groups), Kruskal–Wallis *H* test (three groups), and Pearson’s *χ*^2^ or Fisher’s exact test (categorical data). This study followed the TRIPOD statement (Supplementary Table S1). There were no missing data because of strict criteria requiring complete potassium measurement. Age was dichotomised at 65 years based on clinical evidence and restricted cubic spline analysis (non‑linearity *p* < 0.05). BMI, daily dose and baseline serum potassium were retained as continuous variables after confirming linear logit relationships (all *p* > 0.05 for non‑linearity). The primary cohort was randomly split into 7:3 stratified training and internal validation sets. A total of 1000 bootstrap resamples were conducted to correct overfitting, and optimism-corrected AUC was calculated for model evaluation.

All regression analyses were performed after verifying model assumptions. Univariate logistic regression was conducted in the training set. Variables with *p* < 0.10 were included in the multivariable model [27]. Results were reported as odds ratios (ORs) with 95% confidence intervals (CIs). Collinearity was assessed using the variance inflation factor (VIF). Variables with VIF > 10 were excluded. A prediction nomogram was established using the rms package in R. The optimal cut‑off value was determined by receiver operating characteristic (ROC) curve analysis. Model discrimination was evaluated by the area under the ROC curve (AUC). An AUC > 0.70 was considered acceptable. Calibration was assessed using the Hosmer–Lemeshow test (*p* > 0.05 indicating good calibration), calibration intercept and slope, and calibration plots. Overall performance was measured by the Brier score (< 0.25 acceptable). Decision curve analysis (DCA) was performed in all three cohorts to evaluate the clinical net benefit across risk thresholds of 0–0.3 (0.01 increments). The nomogram was compared with “treat‑all” and “treat‑none” strategies. A sensitivity analysis assessed model robustness using a parsimonious model (Supplementary Table S2). This model included only predictors with *p* < 0.05 from the primary multivariable analysis. Effect estimates remained consistent with the primary model (all candidates with univariate *p* < 0.10).

A two-tailed *p* < 0.05 was considered statistically significant.

### Ethics approval

This study was approved by the Ethics Committee of Xi‘an Central Hospital (No. LW-2025-011). Due to its retrospective design and the use of fully anonymised patient data from electronic medical records without direct intervention, the committee waived the requirement for informed patient consent.

## Results

### Patient enrollment and baseline characteristics

**Fig. 1:**
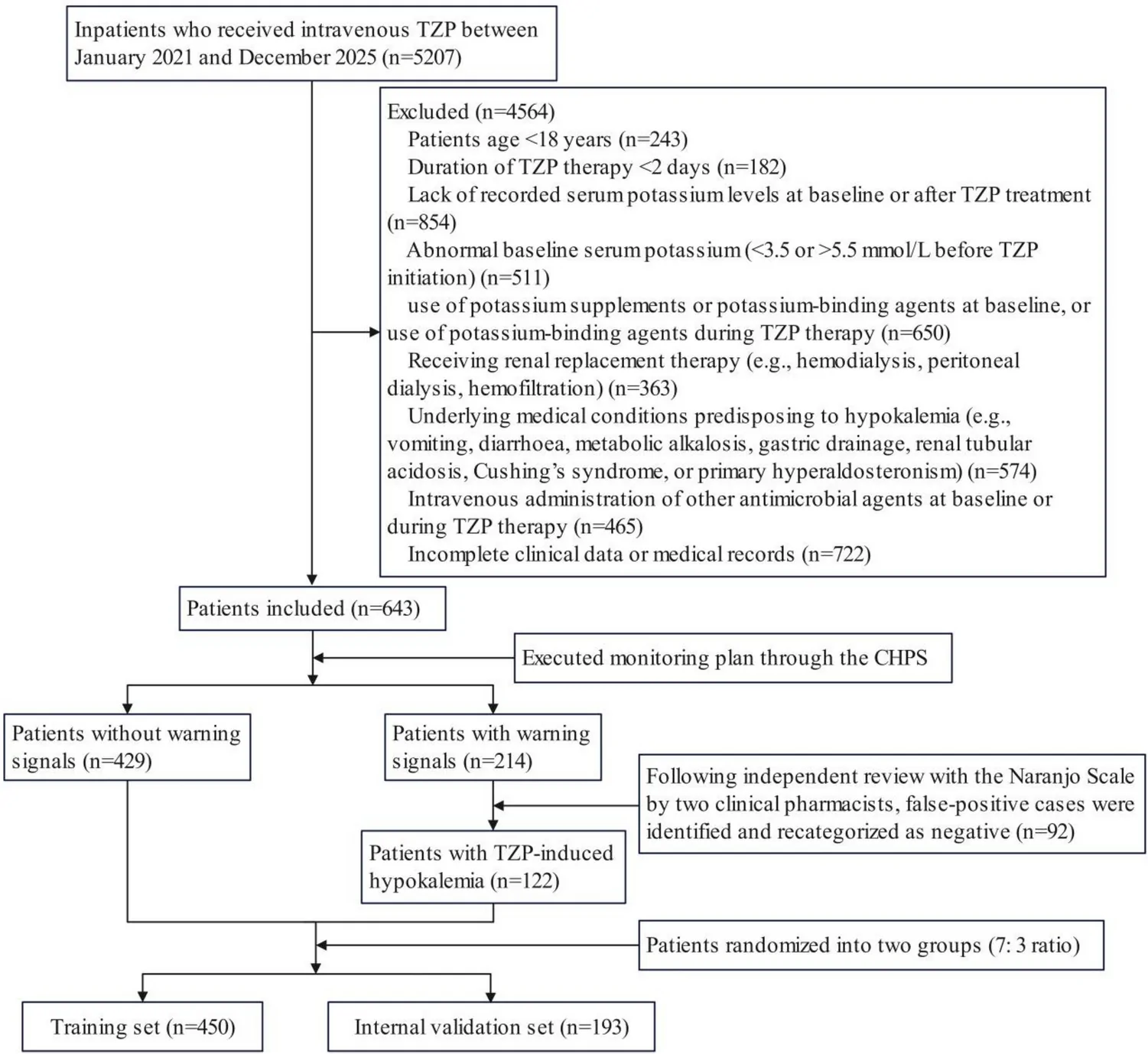
Patient enrollment and exclusion flowchart (January 2021–December 2025). TZP: piperacillin/tazobactam; CHPS: China Hospital Pharmacovigilance System

Between 1 January 2021 and 31 December 2025, 5,207 patients received TZP at Xi’an Central Hospital. After applying the inclusion and exclusion criteria, 4564 patients (87.7%) were excluded, leaving 643 eligible patients (12.3%). Two clinical pharmacists independently assessed 214 CHPS‑generated alerts using the Naranjo scale and confirmed 122 cases of TZP‑induced hypokalaemia. False‑positive cases (n = 92) were reclassified as negative. The 643 patients were then randomised into a training set (n = 450) and an internal validation set (n = 193) in a 7:3 ratio (Fig. 1). The incidence of hypokalaemia was 18.4% (83/450) in the training set and 20.2% (39/193) in the validation set.

Table 1 summarises baseline characteristics. The hypokalaemia and normokalaemia groups were comparable for sex and major comorbidities. However, several factors were significantly associated with hypokalaemia: age ≥ 65 years, lower BMI, ICU admission, lower baseline serum potassium, and greater TZP exposure (daily dose, treatment duration, cumulative dose). Potassium‑wasting diuretics were more common in hypokalaemia patients. Infection sites and other medications showed no consistent subgroup differences. Baseline *p* values are descriptive only and were not adjusted for multiple comparisons.

**Table 1:** Demographic and baseline characteristics in training and internal validation sets

Variables	Total (n = 643)	Training set (n = 450)			Internal validation set (n = 193)		
		Hypokalaemia (n = 83)	Normokalaemia (n = 367)	*p* value	Hypokalaemia (n = 39)	Normokalaemia (n = 154)	*p* value
*Demographi***c**							
Males, n (%)	427 (66.4)	54 (65.1)	249 (67.8)	0.625	26 (66.7)	98 (63.6)	0.724
Age ≥ 65 years, n (%)	423 (65.8)	69 (83.1)	231 (62.9)	< 0.001	34 (87.2)	90 (58.4)	< 0.001
BMI, kg/m^2^, median (IQR)	20.9 (18.6–22.9)	19.0 (17.1–20.1)	21.3 (19.2–23.4)	< 0.001	18.5 (17.7–20.3)	21.5 (18.7–23.5)	< 0.001
Internal medicine, n (%)	364 (56.6)	42 (50.6)	213 (58.0)	0.217	25 (64.1)	84 (54.5)	0.282
Length of hospital stay (days), median (IQR)	14 (10–21)	14 (11–23)	14 (9–20)	0.094	17 (12–21)	14.5 (9–21)	0.086
ICU admission, n (%)	251 (39.0)	57 (68.7)	119 (32.4)	< 0.001	27 (69.2)	48 (31.2)	< 0.001
Surgery, n (%)	368 (57.2)	44 (53.0)	215 (58.6)	0.354	24 (61.5)	85 (55.2)	0.475
*Comorbiditie***s**							
Hypertension, n (%)	322 (50.1)	44 (53.0)	186 (50.7)	0.701	19 (48.7)	73 (47.4)	0.883
Diabetes, n (%)	168 (26.1)	23 (27.7)	98 (26.7)	0.852	10 (25.6)	37 (24.0)	0.834
Cardiovascular disease, n (%)	212 (33.0)	35 (42.2)	108 (29.4)	0.024	19 (48.7)	50 (32.5)	0.059
Cerebrovascular disease, n (%)	226 (35.1)	36 (43.4)	124 (33.8)	0.099	17 (43.6)	49 (31.8)	0.166
Chronic liver disease, n (%)	210 (32.7)	29 (34.9)	114 (31.1)	0.493	15 (38.5)	52 (33.8)	0.582
Chronic kidney disease, n (%)	218 (33.9)	24 (28.9)	124 (33.8)	0.394	16 (41.0)	54 (35.1)	0.489
*Infection site*							
Pneumonia, n (%)	248 (38.6)	38 (45.8)	147 (40.1)	0.338	15 (39.0)	48 (31.2)	0.386
Urinary system, n (%)	201 (31.3)	31 (37.0)	108 (29.4)	0.158	16 (41.0)	46 (29.9)	0.183
Intra-abdominal, n (%)	125 (19.4)	13 (15.7)	72 (19.6)	0.406	10 (25.6)	30 (19.5)	0.397
Skin and soft tissue, n (%)	106 (16.5)	14 (16.9)	59 (16.1)	0.860	7 (17.9)	26 (16.9)	0.875
Sepsis/bacteraemia, n (%)	94 (14.6)	13 (15.7)	54 (14.7)	0.826	5 (12.8)	22 (14.3)	0.814
Bone and joint, n (%)	74 (11.5)	11 (13.3)	42 (11.4)	0.644	3 (7.7)	18 (11.7)	0.669
Others, n (%)	67 (10.4)	8 (9.6)	40 (10.9)	0.737	5 (12.8)	14 (9.1)	0.691
*TZP exposure*							
Daily dose (g), median (IQR)	12 (8–12)	12 (12–12)	8 (8–12)	< 0.001	12 (8–12)	8 (8–12)	0.001
Treatment course (days), median (IQR)	8.5 (6–11.5)	11 (8–13)	8 (6–11)	< 0.001	12 (10–14)	8 (5–11)	< 0.001
Cumulative dose (g), median (IQR)	80 (52–120)	114 (88–150)	72 (48–102)	< 0.001	132 (96–168)	70 (40–105)	< 0.001
*Baseline laboratory parameters*							
Baseline serum potassium (mmol/L), median (IQR)	4.2 (3.8–4.7)	4.0 (3.7–4.2)	4.3 (3.9–4.8)	< 0.001	3.8 (3.7–4.0)	4.3 (3.9–4.8)	< 0.001
Baseline serum calcium (mmol/L), median (IQR)	2.2 (2.1–2.3)	2.2 (2.1–2.3)	2.2 (2.1–2.3)	0.502	2.2 (2.1–2.3)	2.2 (2.1–2.3)	0.452
Baseline serum sodium (mmol/L), median (IQR)	138 (134–141)	138 (133–140)	138 (135–141)	0.180	138 (130–142)	138 (135–141)	0.290
Baseline serum phosphorus (mmol/L), median (IQR)	1.1 (0.9–1.3)	1.0 (0.9–1.1)	1.1 (0.9–1.3)	0.001	1.0 (0.8–1.1)	1.1 (0.9–1.3)	0.002
Total protein (g/L), mean ± SD	63.7 ± 8.6	62.6 ± 8.2	64.1 ± 8.5	0.153	62.3 ± 8.1	63.8 ± 9.0	0.360
Albumin (g/L), mean ± SD	36.2 ± 6.4	35.3 ± 6.3	36.3 ± 6.4	0.196	35.0 ± 6.4	36.8 ± 6.6	0.141
AST (U/L), median (IQR)	23 (17–34)	23 (17–33)	23 (17–34)	0.938	22 (16–33)	23 (16–34)	0.818
ALT (U/L), median (IQR)	17 (11–29)	16 (10–33)	18 (11–29)	0.990	17 (10–26)	16 (10–29)	0.909
Serum creatinine (μmol/L), median (IQR)	71 (54–97)	73 (55–93)	70 (53–95)	0.637	77 (62–97)	72 (55–109)	0.586
Blood urea nitrogen (mmol/L), median (IQR)	6.1 (4.5–9.1)	6.5 (4.7–10.5)	5.9 (4.5–8.9)	0.226	6.0 (4.2–8.6)	6.4 (4.6–10.4)	0.212
Hemoglobin (g/L), median (IQR)	114 (98–130)	116 (99–133)	114 (98–130)	0.699	114 (102–134)	113 (99–128)	0.566
White blood cell count (× 10⁹/L), median (IQR)	7.9 (5.6–11.3)	7.6 (5.4–10.0)	7.9 (5.5–11.4)	0.435	7.4 (4.8–11.6)	8.6 (6.0–12.0)	0.118
Platelet count (× 10⁹/L), median (IQR)	194 (135–264)	196 (152–257)	193 (129–269)	0.740	204 (149–260)	192 (135–263)	0.826
*Concomitant medications*							
Potassium-sparing diuretics, n (%)	64 (10.0)	12 (14.5)	30 (8.2)	0.076	9 (23.1)	13 (8.4)	0.022
NSAIDs, n (%)	109 (17.0)	19 (22.9)	58 (15.8)	0.122	3 (7.7)	29 (18.8)	0.095
β blockers, n (%)	40 (6.2)	4 (4.8)	26 (7.1)	0.455	4 (10.3)	6 (3.9)	0.232
ACEI/ARB, n (%)	49 (7.6)	6 (7.2)	29 (7.9)	0.836	4 (10.3)	10 (6.5)	0.643
Digoxin, n (%)	27 (4.2)	4 (4.8)	14 (3.8)	0.911	1 (2.6)	8 (5.2)	0.786
Potassium-wasting diuretics, n (%)	268 (41.7)	49 (59.0)	143 (39.0)	0.001	23 (59.0)	53 (34.4)	0.005
Insulin, n (%)	101 (15.7)	13 (15.7)	63 (17.2)	0.741	4 (10.3)	21 (13.6)	0.574
Glucocorticoid, n (%)	191 (29.7)	26 (31.3)	107 (29.2)	0.696	10 (25.6)	48 (31.2)	0.501

Daily dose refers to piperacillin content (g/day); TZP is a fixed‑ratio combination (8:1 piperacillin/tazobactam). Data are presented as n (%), median (IQR), or mean ± SD. IQR, Interquartile range; BMI, Body mass index; ICU, Intensive care unit; TZP, Piperacillin/tazobactam; AST, Aspartate aminotransferase; ALT, Alanine aminotransferase; NSAIDs, Non‑steroidal anti‑inflammatory drugs; ACEI, Angiotensin‑converting enzyme inhibitors; ARB: angiotensin II receptor blockers. *p* values are exploratory and were not adjusted for multiple comparisons

### Incidence and severity of hypokalaemia

Among 643 patients, 122 (19.0%) developed TZP‑induced hypokalaemia (Table 2). Mild cases were most common (n = 96, 78.7%), followed by moderate (n = 20, 16.4%) and severe (n = 6, 4.9%). No severe complications occurred after timely intervention. Serum potassium levels at diagnosis and nadir declined significantly with increasing severity (*p* < 0.001 for both). Median serum potassium was 3.20 mmol/L (IQR 3.00–3.33) at diagnosis and 3.10 mmol/L (IQR 2.81–3.30) at nadir. Median time to onset and nadir were both 5.0 days (IQR 3.0–7.0 and 4.0–9.0, respectively), with no significant variation across severity groups (*p* = 0.129 and *p* = 0.178). Severe cases showed non‑significant trends toward longer time to nadir (median 7.5 days) and higher accumulated TZP doses (*p* = 0.062): 159 g vs. 129 g (moderate) and 114 g (mild). Potassium supplementation was significantly associated with severity (*p* = 0.015), occurring in 83.3% of severe, 80.0% of moderate, and 49.0% of mild cases. No significant differences were found in potassium‑wasting diuretics (*p* = 0.105) or glucocorticoids (*p* = 0.349) across severity groups. Given the small number of severe cases (n = 6), *p* values should be interpreted with caution.

**Table 2:** Clinical characteristics of TZP‑induced hypokalaemia stratified by severity

Characteristics	Overall (n = 122)	Severity of hypokalaemia			*p* value
		Mild (n = 96)	Moderate (n = 20)	Severe (n = 6)	
Serum potassium at onset of hypokalaemia (mmol/L), median (IQR)	3.20 (3.00–3.33)	3.26 (3.10–3.37)	2.81 (2.76–2.90)	2.36 (2.32–2.48)	< 0.001
Time to onset (days), median (IQR)	5.0 (3.0–7.0)	4.0 (3.0–7.0)	5.0 (4.0–10.3)	5.5 (5.0–11.0)	0.129
Serum potassium at the nadir of hypokalaemia (mmol/L), median (IQR)	3.10 (2.81–3.30)	3.20 (3.10–3.30)	2.81 (2.76–2.90)	2.31 (2.25–2.35)	< 0.001
Time to nadir of hypokalaemia (days), median (IQR)	5.0 (4.0–9.0)	5.0 (3.3–9.0)	5.0 (4.0–10.3)	7.5 (6.5–14.0)	0.178
Accumulated dose (g), median (IQR)	120.0 (89.5–153.0)	114.0 (88.0–148.5)	129.0 (91.5–169.5)	159.0 (130.5–223.5)	0.062
Potassium supplementation after TZP treatment, n (%)	67 (54.92)	47 (48.96)	16 (80.00)	5 (83.33)	0.015
Combined use of potassium–wasting diuretics, n (%)	72 (59.01)	52 (54.17)	15 (75.00)	5 (83.33)	0.105
Combined use of glucocorticoids, n (%)	36 (29.51)	29 (30.21)	4 (20.00)	3 (50.00)	0.349

Accumulated dose refers to piperacillin content (g/day); TZP: piperacillin/tazobactam; IQR: interquartile ranges. Statistical comparisons involving the severe hypokalaemia subgroup (n = 6) should be interpreted with caution due to limited sample size. p values are provided for descriptive purposes only

### Univariate and multivariable logistic regression analyses

Logistic regression identified independent predictors for TZP‑induced hypokalaemia. Univariate analysis showed ten potential predictors (all *p* < 0.10; Table 3), including age ≥ 65 years, BMI, hospital stay, ICU admission, cardiovascular disease, daily dose, baseline serum potassium, baseline serum phosphorus, and potassium‑sparing or potassium‑wasting diuretics. Cumulative dose and treatment course were excluded due to high collinearity with daily dose (VIF: 6.382, 19.054, and 25.914). Multivariable analysis of the 10 candidates identified five independent predictors: age ≥ 65 years (OR = 2.118, 95% CI 1.003–4.472, *p* = 0.049), BMI (OR = 0.671, 95% CI 0.591–0.762, *p* < 0.001), ICU admission (OR = 2.915, 95% CI 1.504–5.651, *p* = 0.002), daily dose (OR = 1.280, 95% CI 1.110–1.476, *p* < 0.001), and baseline serum potassium (OR = 0.123, 95% CI 0.056–0.271, *p* < 0.001). Hospital stay length, cardiovascular disease, baseline serum phosphorus, and diuretic use were not significant (all *p* > 0.05; Table 3).

**Table 3:** Logistic regression analyses of factors associated with TZP‑induced hypokalaemia (training set, n = 450)

Variables	Univariate		Multivariable	
	OR (95% CI)	*p* value	OR (95% CI)	*p* value
Age (≥ 65 years)	2.902 (1.573–5.353)	0.001	2.118 (1.003–4.472)	0.049
BMI (kg/m^2^)	0.701 (0.632–0.778)	< 0.001	0.671 (0.591–0.762)	< 0.001
Length of hospital stay (days)	1.023 (1.006–1.040)	0.009	1.001 (0.981–1.021)	0.934
ICU admission	4.569 (2.736–7.629)	< 0.001	2.915 (1.504–5.651)	0.002
Cardiovascular disease	1.749 (1.071–2.855)	0.025	1.391 (0.722–2.679)	0.324
Daily dose (g)	1.261 (1.123–1.416)	< 0.001	1.280 (1.110–1.476)	< 0.001
Baseline serum potassium (mmol/L)	0.164 (0.088–0.307)	< 0.001	0.123 (0.056–0.271)	< 0.001
Baseline serum phosphorus (mmol/L)	0.337 (0.153–0.746)	0.007	0.701 (0.265–1.858)	0.476
Potassium-sparing diuretics	1.899 (0.927–3.888)	0.080	2.142 (0.777–5.904)	0.141
Potassium-wasting diuretics	2.258 (1.390–3.667)	0.001	1.152 (0.574–2.313)	0.691

Daily dose refers to piperacillin content (g/day). TZP is a fixed‑ratio combination (8:1 piperacillin/tazobactam). Cumulative dose and treatment course were excluded from multivariable analysis due to high collinearity with daily dose (variance inflation factor, VIF: daily dose 6.382, cumulative dose 19.054, treatment course 25.914); TZP, Piperacillin/tazobactam; OR, Odds ratio; CI, Confidence Interval; BMI, Body mass index; ICU, Intensive care unit

### Sensitivity analyses

A sensitivity analysis was performed to assess model robustness given the events per variable (EPV) ratio. A parsimonious model was constructed using only the five predictors with *p* < 0.05 from the primary analysis. As shown in Supplementary Table S2, effect estimates remained largely unchanged, indicating robustness against overfitting. This five‑predictor model was adopted as the final nomogram, achieving an EPV of 16.6 (83/5)—well within the recommended range.

### External validation and cohort comparison

The external validation cohort included 141 patients from Tongchuan People’s Hospital, of whom 27 (19.1%) developed TZP‑induced hypokalaemia. Baseline characteristics of the derivation cohort (Xi’an, n = 643) and external cohort (Tongchuan, n = 141) are compared in Supplementary Table S3. The two cohorts were generally comparable in age, sex, ICU admission, daily dose, and baseline serum potassium (all *p* > 0.05). However, the external cohort had lower BMI, shorter hospital stay, and higher AST and ALT levels (all *p* < 0.05), likely reflecting variations in case mix and clinical practices between centres.

### Nomogram development and validation

**Fig. 2:**
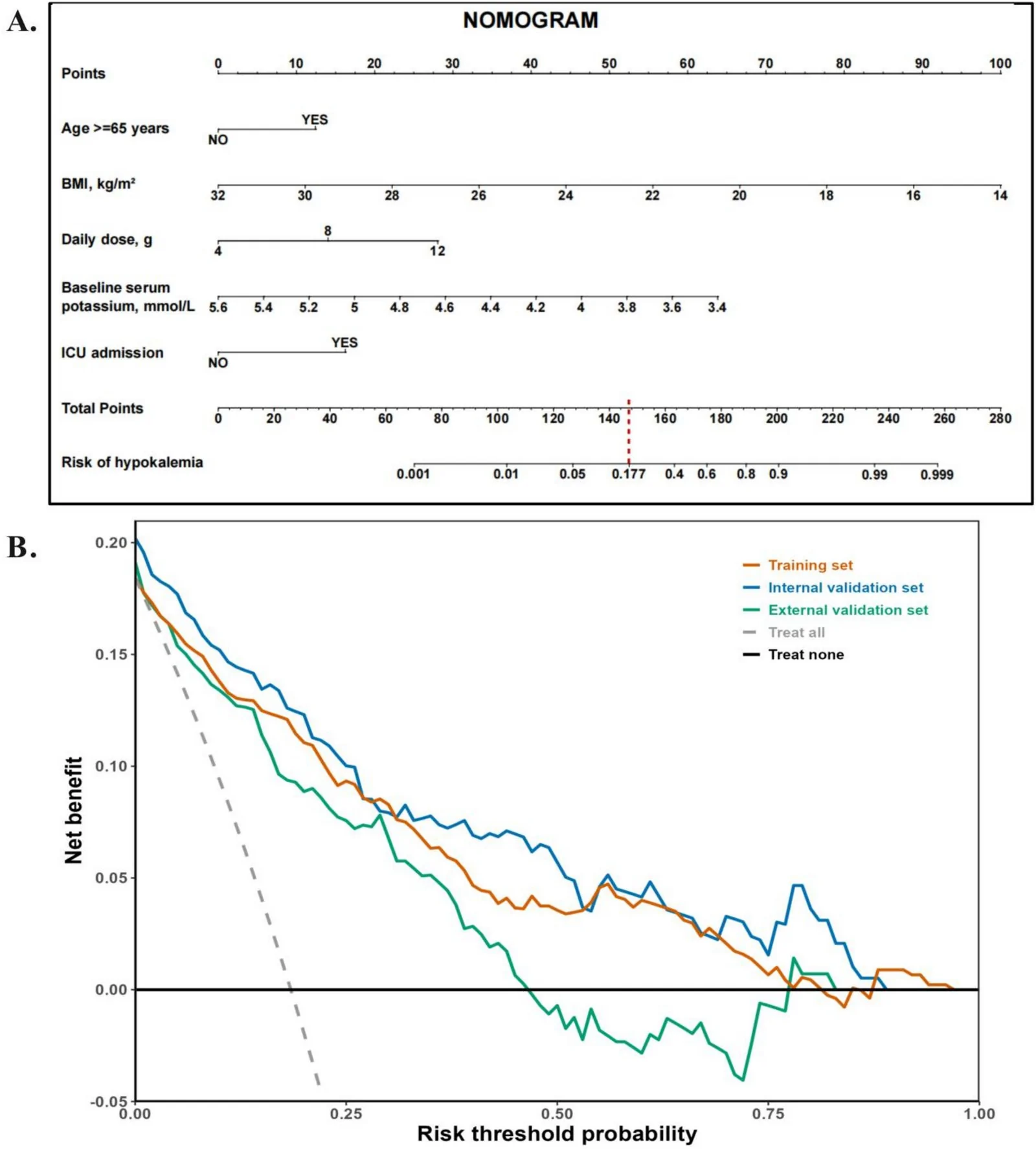
Nomogram for predicting the risk of TZP‑induced hypokalaemia and decision‑curve analysis for model validation. **A** Nomogram for individualised risk prediction. BMI, body mass index; ICU, intensive care unit; TZP, piperacillin/tazobactam. Daily dose refers to piperacillin content (grams per day). TZP is a fixed‑ratio combination (8:1 piperacillin/tazobactam). To use the nomogram, locate the value of each predictor on its corresponding axis, draw a vertical line to the “Points” axis to obtain the points for each predictor, sum the points, locate the total points on the “Total Points” axis, and draw a vertical line to the “Risk of TZP‑induced hypokalaemia” axis to obtain the predicted probability. A risk threshold of 0.177 was identified as the optimal cut‑off value. **B** Decision‑curve analysis for the derivation and validation cohorts. The graph shows net benefit (y‑axis) as a function of risk threshold probability (x‑axis). Blue curve: training set; red curve: internal validation set; green curve: external validation set; gray dashed line: “treat‑all” strategy; gray solid line: “treat‑none” strategy (net benefit = 0)

**Fig. 3:**
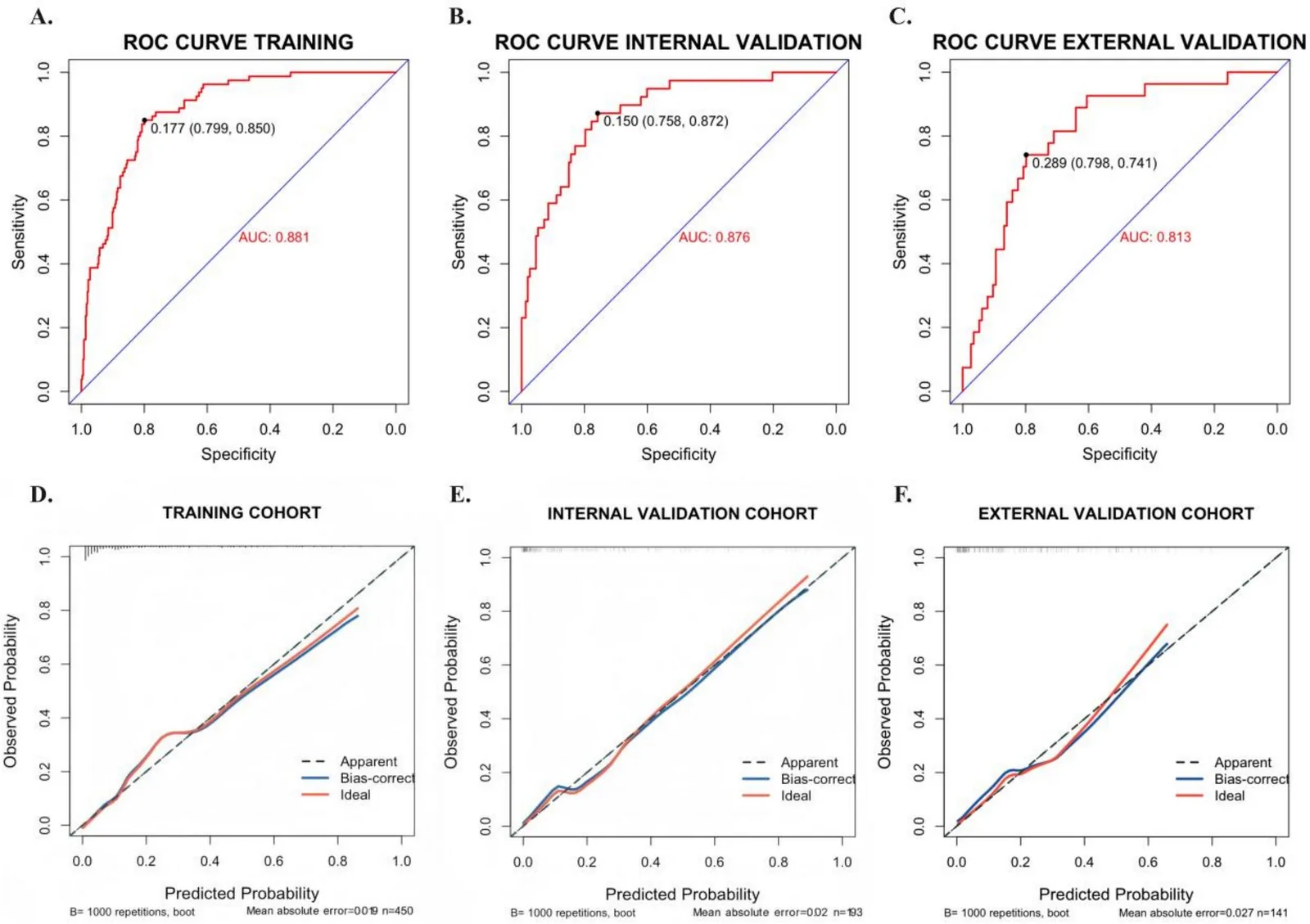
Performance of the nomogram in the training, internal validation, and external validation sets. **A–C** ROC curves with AUC values of 0.881, 0.876, and 0.813, respectively. **D–F** Calibration plots with Hosmer–Lemeshow test p-values of 0.422, 0.630, and 0.351, respectively. ROC, receiver operating characteristic; AUC, area under the curve

Based on the five-predictor model (Supplementary Table S2), a nomogram was constructed (Fig. 2A). The prediction equation is: ln(*p*/(1 − *p*)) = 11.121 + 0.870 × Age (≥ 65 years) − 0.385 × BMI + 1.134 × ICU admission + 0.255 × Daily dose (g) − 2.104 × Baseline serum potassium (mmol/L). The optimism-corrected AUC was 0.870, close to the apparent AUC of 0.881, indicating minimal overfitting. The nomogram showed good discrimination: AUC 0.881 (95% CI 0.846–0.917) in the training set and 0.876 (95% CI 0.817–0.935) in the internal validation set (Fig. 3A, B). The optimal cut‑off was 0.177. An Excel risk calculator (Supplementary Table S4) is provided for bedside use. At this threshold, the training set achieved an accuracy of 80.9%, sensitivity of 85.0% and specificity of 79.9%. The internal validation set achieved an accuracy of 78.6%, sensitivity of 82.1% and specificity of 77.8%. Calibration was good (Hosmer‑Lemeshow* p* = 0.422 and 0.630; Fig. 3D, E).In the external validation cohort (n = 141, 27 events), the nomogram achieved an AUC of 0.813 (95% CI 0.728–0.897), an accuracy of 78.7%, a sensitivity of 74.1% and a specificity of 79.8% (Fig. 3C). The calibration curve showed good agreement (Hosmer‑Lemeshow *p* = 0.351; Fig. 3F). Calibration intercept/slope and Brier score further evaluated performance (Supplementary Table S5). Calibration intercepts were 0.000, 0.126 and − 0.402; slopes were 1.000, 0.962 and 0.759. Brier scores were 0.099, 0.105 and 0.127 (all < 0.25). DCA showed positive net benefit at thresholds 0.10–0.70 for the derivation cohorts and 0.10–0.48 for the external cohort (Fig. 2B).

## Discussion

This study investigated the incidence, risk factors, severity and predictive nomogram for TZP‑induced hypokalaemia in hospitalised adults. The incidence was 19.0% in 643 patients, consistent with recent real‑world findings (17.9%–24.8%) [18, 19] but higher than early clinical trial results (1.5%–12.6%) [16, 17], reflecting routine practice with elderly and comorbid patients. This confirms that TZP‑associated hypokalaemia is common, not rare. Pharmacovigilance evidence supports an association between TZP and kaliuretic effects, independent of infection type or baseline renal function [13].

The median diagnostic potassium was 3.20 mmol/L (nadir 3.10 mmol/L), above previous reports [20]. The median time to onset and nadir was 5.0 days, in line with prior studies [19, 20]. Severe cases showed a non‑significant trend toward delayed nadir, suggesting that severe hypokalaemia may be related to continuous potassium depletion [28]. The temporal pattern (peak day 5, 3–7 day window) aligns with international findings [14]. Most cases were mild (78.7%), with moderate (16.4%) and severe (4.9%) cases differing from previous severity distributions [19], possibly due to varied monitoring methods. Hypokalaemia severity correlated with potassium supplementation demand (*p* = 0.015). The supplementation rate was 83.3% in severe cases and 80.0% in moderate cases, suggesting most moderate and severe TZP‑induced hypokalaemia requires clinical intervention.

A five‑predictor nomogram (advanced age, low BMI, ICU admission, high daily dose, low baseline potassium) showed good discrimination: AUC 0.881 (training), 0.876 (internal validation) and 0.813 (external validation). Calibration was good across all cohorts (Hosmer‑Lemeshow *p* > 0.05). Brier scores were < 0.25. DCA confirmed positive net benefit at clinically relevant thresholds (0.10–0.70 for derivation cohorts, 0.10–0.48 for the external cohort), supporting potential clinical utility pending further validation.

Age ≥ 65 years was an independent predictor, consistent with prior reports [19, 20]. Age‑related decline in renal function may impair potassium homeostasis and reduce adaptive capacity to kaliuretic stress [29]. Older patients often have multiple comorbidities and use diuretics or other potassium‑wasting agents, which may further increase susceptibility [30, 31]. Lower BMI was associated with higher risk, aligning with previous findings [19]. Patients with lower BMI may have reduced total body potassium reserves and less lean muscle mass (the main intracellular potassium store), which may be associated with greater vulnerability to kaliuretic stressors [32, 33]. ICU admission was independently associated with higher risk. Critically ill patients may experience inflammation‑mediated effects on renal tubules [34], polypharmacy with kaliuretic agents (e.g., loop diuretics), haemodynamic instability, and stress‑induced dysregulation of aldosterone and cortisol. All of these factors may promote potassium wasting [9, 35]. Daily dose was a robust predictor (OR = 1.280 per 1 g/day increase). A non‑significant trend toward higher accumulated doses in more severe cases suggests a possible dose‑response relationship. This is mechanistically plausible, as piperacillin acts as a non‑reabsorbable anion, enhancing distal nephron sodium delivery and potassium secretion [36, 37]. Higher baseline serum potassium was protective, consistent with reports for other penicillins [38, 39]; lower pre‑treatment potassium reserves reduce the capacity to buffer renal losses. These five predictors align with global consensus: elderly and ICU patients have impaired compensatory capacity and multiple potassium‑wasting exposures; lower baseline potassium is protective; higher daily dose drives dose‑dependent renal excretion [13].

A key strength is the combination of CHPS‑based automated screening with full manual review of all laboratory data. This minimised outcome ascertainment bias and captured false‑negative cases. The nomogram, with rigorous internal and external validation, shows good discrimination and calibration. Severity‑stratified intervention rates provide real‑world data supporting hierarchical monitoring. To enhance clinical utility, an Excel‑based calculator (Supplementary Table S4) is available for instant risk estimation without manual calculation.

Several limitations warrant consideration. First, the retrospective design may have residual confounding. Uncontrolled factors include ICU admission (haemodynamic instability, polypharmacy), diuretic use (regimen changes not captured), renal function fluctuations, infection severity (no validated scoring system), and treatment duration (collinear with daily dose). Second, the nomogram is based on baseline variables only and does not capture dynamic changes during therapy. Third, serum magnesium was not routinely monitored, despite its deficiency being common in hypokalaemia [40]. Fourth, strict exclusion criteria removed 87.7% of the initial cohort. While necessary to isolate TZP‑specific effects, this limits generalisability to patients receiving TZP as monotherapy who are normokalaemic at baseline and free from major potassium‑modifying interventions. The lower AUC in the external cohort (0.813 vs. 0.881) may reflect expected performance degradation in a distinct population. The calibration slope of 0.759 suggests some overfitting, indicating that model recalibration may be needed before clinical implementation. Future prospective studies should consider intercept or slope recalibration to optimise local performance. Finally, although externally validated, the model requires prospective evaluation in diverse multicentre settings with time‑varying confounders to confirm its clinical utility.

## Conclusion

TZP‑induced hypokalaemia is common in hospitalised adults. A nomogram based on five clinical variables (age ≥ 65 years, BMI, ICU admission, daily dose and baseline serum potassium) was developed and externally validated. This tool provides a potential approach for individualised risk assessment at the point of care and may assist in the early identification of at‑risk patients to guide targeted electrolyte monitoring. Nevertheless, prospective validation is required before widespread clinical implementation.

## Supplementary Information

Below is the link to the electronic supplementary material.

Supplementary file1 (DOCX 22 KB)

Supplementary file2 (DOCX 13 KB)

Supplementary file3 (DOCX 21 KB)

Supplementary file4 (XLSX 12 KB)

Supplementary file5 (DOCX 13 KB)

## Data Availability

All original baseline data will be provided as attached DATA files, and all analysed data are available in the manuscript and supplementary files. The relevant software code is available from the corresponding author on reasonable request. The Funding and Competing Interests sections are retained without revision.
