# Supplementary material for: Active surveillance and risk prediction of piperacillin/tazobactam‑associated hypokalaemia: development and external validation of a clinical nomogram
Source: Int J Clin Pharm. 2026 Jul 24;48(5):2105–16. doi: 10.1007/s11096-026-02195-z (PMC13569512; doi:10.1007/s11096-026-02195-z)
Supplement: Supplementary file 1 [file 11096_2026_2195_MOESM1_ESM.docx]

****Supplementary Table S1.** TRIPOD checklist for prediction model development and validation.**

| **Section/Topic** | **Item** | **Recommendation** | **Reported on Page/Line** |
| --- | --- | --- | --- |
| **Title and Abstract** |  |  |  |
| Title | 1 | Identify the study as developing and/or validating a multivariable prediction model | Title page |
| Abstract | 2 | Provide a summary of objectives, study design, setting, participants, sample size, predictors, outcome, statistical analysis, results, and conclusions | Abstract |
| **Introduction** |  |  |  |
| Background | 3a | Explain the medical context and rationale for developing the model | Introduction, para 1-4 |
| Objectives | 3b | Specify the objectives, including whether the study describes development and/or validation of the model | Introduction, Aim |
| **Methods** |  |  |  |
| Source of data | 4a | Describe the study design or source of data (e.g., RCT, cohort) | Methods: Study design and patient cohort |
| Key dates | 4b | Specify key study dates, including start of accrual, end of accrual, and follow-up | Methods: Study design and patient cohort |
| Participants | 5a | Specify the eligibility criteria for participants | Methods: Inclusion/exclusion criteria |
| Participants | 5b | Describe any treatment received before outcome measurement | Methods: Information collection |
| Outcome | 6a | Clearly define the outcome, including how and when assessed | Methods: Study design |
| Outcome | 6b | Report any actions to blind assessment of the outcome to predictors | Methods: CHPS + independent pharmacist review |
| Predictors | 7a | Clearly define all predictors used in the model | Methods: Information collection |
| Predictors | 7b | Report any actions to blind assessment of predictors to the outcome | N/A (retrospective design) |
| Sample size | 8 | Explain how the sample size was determined | Methods: Consecutive sampling; EPV discussed |
| Missing data | 9 | Describe how missing data were handled | No missing data due to strict inclusion criteria requiring complete potassium measurements |
| Statistical analysis methods | 10a | Describe how predictors were handled in the analyses (e.g., coding, continuous vs. categorical) | Age dichotomised at 65y; BMI, daily dose, baseline K⁺ as continuous; linearity verified by splines |
| Statistical analysis methods | 10b | Specify the type of model (e.g., logistic regression) | Methods: Statistical analysis |
| Statistical analysis methods | 10c | Describe how predictors were selected for inclusion in the model | Methods: Univariate *p*<0.10 → multivariable; VIF<10 |
| Statistical analysis methods | 10d | Describe how the model was developed (e.g., which predictors retained, method for model building) | Methods: Multivariable logistic regression → parsimonious 5-variable model |
| Risk groups | 11 | Describe how the model was validated (e.g., internal validation using split-sample, bootstrap) | 7:3 split for internal validation; 1,000 bootstrap resamples for optimism correction; external validation at Tongchuan People's Hospital |
| Performance measures | 12 | Specify all measures used to assess model performance (discrimination and calibration) | Methods: Statistical analysis (AUC for discrimination, Hosmer-Lemeshow for calibration) |
| Model updating | 13 | Describe any model updating during validation | N/A |
| **Results** |  |  |  |
| Participants | 14a | Describe the flow of participants through the study | Results: Patient enrollment; Figure 1 |
| Participants | 14b | Describe the characteristics of the participants | Results: Table 1 |
| Participants | 14c | Describe the number of participants with missing data for each predictor | No missing data |
| Outcome events | 15 | Report the number of outcome events and number of participants | Results: 122 events among 643 participants (18.97%) |
| Model specification | 16a | Present the full prediction model (regression coefficients, intercept) | Results: Equation: ln(*p*/(1-*p*)) = 11.121 + 0.870×Age(≥65y) − 0.385×BMI + 1.134×ICU + 0.255×Daily dose − 2.104×Baseline serum potassium |
| Model performance | 16b | Present the model's performance (discrimination and calibration) with confidence intervals | Results: AUC 0.881 (0.846–0.917); H-L *p*=0.422; Figure 3 |
| Model updating | 17 | Report any model updating during validation | N/A |
| **Discussion** |  |  |  |
| Limitations | 18 | Discuss limitations of the study | Discussion: Strengths and limitations |
| Interpretation | 19 | Provide an overall interpretation of the results | Discussion: Conclusions |
| Implications | 20 | Discuss the clinical implications and future research directions | Discussion: Conclusions |
| **Other Information** |  |  |  |
| Funding | 21 | Provide the source of funding and conflicts of interest | Funding statement; Competing interests |
