# Supplementary material for: Active surveillance and risk prediction of piperacillin/tazobactam‑associated hypokalaemia: development and external validation of a clinical nomogram
Source: Int J Clin Pharm. 2026 Jul 24;48(5):2105–16. doi: 10.1007/s11096-026-02195-z (PMC13569512; doi:10.1007/s11096-026-02195-z)
Supplement: Supplementary file 2 [file 11096_2026_2195_MOESM2_ESM.docx]

****Supplementary Table S2.** Comparison of effect estimates between the primary 10‑variable model and the parsimonious 5‑variable model for predicting TZP‑induced hypokalaemia.**

| **Variable** | **Primary model (10 variables)** | | | **Parsimonious model (5 variables)** | | |
| --- | --- | --- | --- | --- | --- | --- |
|  | **B (coefficient)** | **OR (95% CI)** | ***p* value** | **B (coefficient)** | **OR (95% CI)** | ***p* value** |
| **Age (≥65 years)** | **0.750** | **2.118 (1.003–4.472)** | **0.049** | **0.870** | **2.386 (1.169–4.870)** | **0.017** |
| **BMI (kg/m²)** | **-0.399** | **0.671 (0.591–0.762)** | **<0.001** | **-0.385** | **0.680 (0.602–0.769)** | **<0.001** |
| **ICU admission** | **1.070** | **2.915 (1.504–5.651)** | **0.002** | **1.134** | **3.108 (1.694–5.704)** | **<0.001** |
| **Daily dose (g)** | **0.247** | **1.280 (1.110–1.476)** | **<0.001** | **0.255** | **1.291 (1.122–1.485)** | **<0.001** |
| **Baseline serum potassium (mmol/L)** | **-2.094** | **0.123 (0.056–0.271)** | **<0.001** | **-2.104** | **0.122 (0.057–0.263)** | **<0.001** |

**Note: The parsimonious model included only the five variables that were statistically significant in the primary model. OR, odds ratio; CI, confidence interval; BMI, body mass index; ICU, intensive care unit.**
