# Supplementary material for: Active surveillance and risk prediction of piperacillin/tazobactam‑associated hypokalaemia: development and external validation of a clinical nomogram
Source: Int J Clin Pharm. 2026 Jul 24;48(5):2105–16. doi: 10.1007/s11096-026-02195-z (PMC13569512; doi:10.1007/s11096-026-02195-z)
Supplement: Supplementary file 3 [file 11096_2026_2195_MOESM3_ESM.docx]

****Supplementary Table S3.**** Comparison of Dervation and External validation cohorts.

| Variables | Derivation cohort (Xi’an n=643) | External validation cohort  (Tongchuan n=141) | *p* value |
| --- | --- | --- | --- |
| **Demographic** |  |  |  |
| Males, n(% ) | 427(66.4) | 94(66.7) | 0.953 |
| ≥65, n(% ) | 423(65.8) | 90(63.8) | 0.658 |
| Median BMI, Kg/m^2^ (IQR) | 20.9(18.6-22.9) | 19.7(18.4-22) | 0.047 |
| Internal medicine, n (%) | 364(56.6) | 71(50.4) | 0.176 |
| Median length of hospital stay, days (IQR) | 14(10-21) | 11(8-14) | ＜0.001 |
| ICU admission, n (%) | 251(39) | 49(34.8) | 0.343 |
| Surgery, n (%) | 368(57.2) | 82(58.2) | 0.841 |
| **Comorbidities** |  |  |  |
| Hypertension, n (%) | 322(50.1) | 60(42.6) | 0.105 |
| Diabetes, n (%) | 168(26.1) | 32(22.7) | 0.397 |
| Cardiovascular disease, n (%) | 212(33) | 49(34.8) | 0.684 |
| Cerebrovascular disease, n (%) | 226(35.1) | 53(37.6) | 0.584 |
| Chronic liver disease, n (%) | 210(32.7) | 47(33.3) | 0.877 |
| Chronic kidney disease, n (%) | 218(33.9) | 53(37.6) | 0.405 |
| **Infection site** |  |  |  |
| Pneumonia, n (%) | 248(38.6) | 47(33.3) | 0.245 |
| Urinary system, n (%) | 201(31.3) | 37(26.2) | 0.241 |
| Intra-abdominal, n (%) | 125(19.4) | 24(17) | 0.507 |
| Skin and soft tissue, n (%) | 106(16.5) | 24(17) | 0.877 |
| Sepsis/bacteraemia, n (%) | 94(14.6) | 21(14.9) | 0.933 |
| Bone and joint, n (%) | 74(11.5) | 16(11.3) | 0.957 |
| Others, n (%) | 67(10.4) | 14(9.9) | 0.862 |
| **TZP exposure** |  |  |  |
| Median daily dose, g (IQR) | 12(8-12) | 12(8-12) | 0.125 |
| Median treatment course, days (IQR) | 8.5(6-11.5) | 9(7-12) | 0.380 |
| Median cumulative doses, g (IQR) | 80(52-120) | 84(56-120) | 0.234 |
| Median cumulative DDDs (IQR) | 5.7(3.7-8.6) | 6(4-8.6) | 0.234 |
| **Laboratory tests** |  |  |  |
| Median serum potassium, mmol/L (IQR) | 4.2(3.8-4.7) | 4.2(3.9-4.6) | 0.538 |
| Median serum calcium, mmol/L (IQR) | 2.2(2.1-2.3) | 2.2(2.1-2.4) | 0.133 |
| Median serum sodium, mmol/L (IQR) | 138(134-141) | 137.6(133.7-142.1) | 0.673 |
| Median serum phosphorus, mmol/L (IQR) | 1.1(0.9-1.3) | 1.1(1-1.3) | 0.105 |
| Median total protein, g/L (IQR) | 63.7±8.6 | 64.4±8.3 | 0.404 |
| Median albumin, g/L (IQR) | 36.2±6.4 | 37.1±6.5 | 0.142 |
| Median AST, U/L (IQR) | 23(17-34) | 29.2(21.5-35.8) | 0.002 |
| Median ALT, U/L (IQR) | 17(11-29) | 30.6(19-40.2) | ＜0.001 |
| Median Scr, μmol/L (IQR) | 71(54-97) | 74.7(57.5-87) | 0.945 |
| Median BUN, mmol/L (IQR) | 6.1(4.5-9.1) | 6.9(5.2-8.1) | 0.087 |
| Median Hb, g/L (IQR) | 114.6±24.5 | 113.5±7.7 | 0.589 |
| Median WBC, ×10^9^/L (IQR) | 7.9(5.6-11.3) | 8.7(5.4-13.1) | 0.118 |
| Median PLT, ×10^9^/L (IQR) | 194(135-264) | 199(155.5-252.5) | 0.218 |
| **Concomitant medications, n (%)** |  |  |  |
| Potassium-sparing diuretics, n (%) | 64(10) | 9(6.4) | 0.186 |
| NSAIDs, n (%) | 109(17) | 26(18.4) | 0.672 |
| β blockers, n (%) | 40(6.2) | 3(2.1) | 0.053 |
| ACEI/ARB, n (%) | 49(7.6) | 14(9.9) | 0.361 |
| Digoxin, n (%) | 27(4.2) | 10(7.1) | 0.142 |
| Potassium-wasting diuretics, n (%) | 268(41.7) | 48(34) | 0.094 |
| Insulin, n (%) | 101(15.7) | 20(14.2) | 0.650 |
| Glucocorticoid, n (%) | 191(29.7) | 40(28.4) | 0.753 |

Daily dose refers to piperacillin content (g/day); TZP is a fixed‑ratio combination (8:1 piperacillin/tazobactam). Data are presented as n (%), median (IQR), or mean ± SD. IQR, interquartile range; BMI, body mass index; ICU, intensive care unit; TZP, piperacillin/tazobactam; DDDs, defined daily doses; AST, aspartate aminotransferase; ALT, alanine aminotransferase; Scr, serum creatinine; BUN, blood urea nitrogen; WBC, white blood cell; Hb, hemoglobin; PLT, platelet; NSAIDs, non-steroidal antiinflammatory drugs; ACEI, angiotensin-converting enzyme inhibitors; ARB, angiotensin II receptor blockers.
