# Supplementary material for: Active surveillance and risk prediction of piperacillin/tazobactam‑associated hypokalaemia: development and external validation of a clinical nomogram
Source: Int J Clin Pharm. 2026 Jul 24;48(5):2105–16. doi: 10.1007/s11096-026-02195-z (PMC13569512; doi:10.1007/s11096-026-02195-z)
Supplement: Supplementary file 4 [file 11096_2026_2195_MOESM5_ESM.docx]

****Supplementary Table S5.** Extended model performance metrics across derivation and validation cohorts.**

| Metric | Training set (n=450) | Internal validation set (n=193) | External validation set (n=141) |
| --- | --- | --- | --- |
| **Calibration** | | | |
| Calibration intercept | 0.000 | 0.126 | −0.402 |
| Calibration slope | 1.000 | 0.962 | 0.759 |
| Hosmer‑Lemeshow *p* value | 0.422 | 0.630 | 0.351 |
| **Overall performance** | | | |
| Brier score | 0.099 | 0.105 | 0.127 |
| **Clinical utility** | | | |
| DCA net benefit threshold range | 0.10–0.70 | 0.10–0.70 | 0.10–0.48 |

**DCA, decision‑curve analysis.**
